# Non-antibiotic-Associated Petechial Rash in an Infectious Mononucleosis-Positive Patient: A Case Report

**DOI:** 10.7759/cureus.82622

**Published:** 2025-04-20

**Authors:** Leah Cliatt, Malgorzata Zembrzuska, Julia Felsenheld, Isha Suthar

**Affiliations:** 1 Dermatology, Rowan-Virtua School of Osteopathic Medicine, Stratford, USA; 2 Psychiatry, Rowan-Virtua School of Osteopathic Medicine, Stratford, USA; 3 Family Medicine, Rowan-Virtua School of Osteopathic Medicine, Stratford, USA

**Keywords:** antibiotic rash, ebv, infectious mononucleosis, itp, petechial rash

## Abstract

Epstein-Barr virus (EBV) is the primary cause of infectious mononucleosis (IM). A cutaneous manifestation is typically observed after the administration of penicillin. We present a case of an otherwise healthy 18-year-old male patient with IM who developed a peripheral cutaneous petechial rash despite no recent exposure to penicillin. He exhibited classic signs of IM, including fever, fatigue, and pharyngitis. Ultrasound imaging demonstrated splenomegaly. He was confirmed to have IM via EBV antigen testing. Additional evaluation returned negative results for *Chlamydia trachomatis*, *Neisseria gonorrhoeae*, cytomegalovirus (CMV), human immunodeficiency virus (HIV), and *Treponema pallidum.* A complete blood count was within normal limits, effectively ruling out other potential causes of the rash. The rash was attributed to IM and resolved spontaneously within 10 days without treatment. In the absence of drug exposure or co-infection, an autoimmune mechanism may be implicated. This case underscores the need for further investigation into immune-mediated processes and suggests that the pathogenesis of IM may be more complex than previously understood.

## Introduction

Infectious mononucleosis (IM) is a common viral syndrome caused by the Epstein-Barr virus (EBV), transmitted primarily through infected saliva. It predominantly affects adolescents and young adults between the ages of 15 and 24 [1,2]. IM commonly presents with fever, fatigue, pharyngitis, and posterior cervical lymphadenopathy. Splenomegaly may be detected on physical exam. Palatal petechiae can be observed; however, a cutaneous petechial rash is uncommon. Petechiae are small, non-blanching hemorrhagic lesions resulting from capillary leakage and may indicate conditions such as thrombocytopenia, vasculitis, or disseminated intravascular coagulation (DIC) [3]. While a petechial rash can occur in mononucleosis, a severe and widespread petechial rash in the absence of medication exposure is exceedingly rare [4].

EBV is a herpesvirus with an estimated global prevalence of approximately 90% [5,6]. While EBV is primarily transmitted via oral secretions, it can also be spread through blood transfusions and organ transplantations [7]. EBV primarily affects adolescents, with seroprevalence increasing with age. In the United States, EBV seroprevalence is approximately 50% in children aged 6-8 years, rising to 89% in individuals aged 18-19. Additionally, socioeconomic factors, such as race/ethnicity and household income, are associated with increased EBV seroprevalence, with higher rates observed among minority and lower-income populations [8,9]. EBV establishes latency in B cells and reactivates later in life, contributing to the development of nasopharyngeal carcinoma, lymphomas, and autoimmune diseases [7]. 

Laboratory findings in IM often include lymphocytosis with atypical lymphocytes. These lymphocytes, or Downey cells, are antigenically stimulated mature T cells that are larger, have a lower nuclear-to-cytoplasm ratio, and have an indented or folded nucleus [2]. The Monospot test detects heterophile antibodies and has a sensitivity of 87% and a specificity of 91% [10]. EBV-specific antibody testing can identify viral capsid antigen (VCA)-IgM, indicative of an active infection, while the presence of VCA-IgG or Epstein-Barr nuclear antigen (EBNA) IgG suggests past infection with EBV [1].

Treatment is typically supportive and includes hydration, antipyretics, analgesics, and rest. Patients are advised to avoid contact sports or strenuous activity due to the risk of splenic rupture. As IM is a self-limiting illness, antiviral or corticosteroid therapy is generally not necessary [10].

## Case presentation

This case involves an 18-year-old male patient with no significant past medical history who presented to the clinic with a rash on his bilateral arms and legs. The patient reported a diagnosis of *Streptococcus pyogenes* infection one month prior and had been seen in the office for an antibiotic change from clindamycin to azithromycin due to a history of small bowel obstruction (SBO). He recovered from the streptococcal infection but developed a recurrent sore throat and fever 3-4 days prior to presenting to the clinic. He subsequently visited an urgent care center, where he tested positive for IM via a rapid Monospot test. At the time of the clinic evaluation, he reported a severe sore throat that was interfering with sleep.

On the same day his sore throat recurred, the patient began noticing a petechial rash that initially appeared on his mid-thighs and progressively worsened over four days, eventually extending to his bilateral legs and arms. The rash was most pronounced on his lower legs bilaterally. He reported associated burning and mild pruritus. On the second day of the rash, he experienced significant discomfort, including a painful bump on the sole of his right foot, which caused difficulty ambulating. This was resolved by the time of his clinic visit. He had attempted treatment with cortisone cream and Benadryl, both of which were ineffective. Additionally, the patient reported abdominal pain and a sensation of pressure in the right upper quadrant (RUQ) and left upper quadrant (LUQ) that began the morning of the clinic visit. He denied fever, chills, abdominal pain, nausea, vomiting, diarrhea, urinary symptoms, or jaundice. 

**Figure 1:**
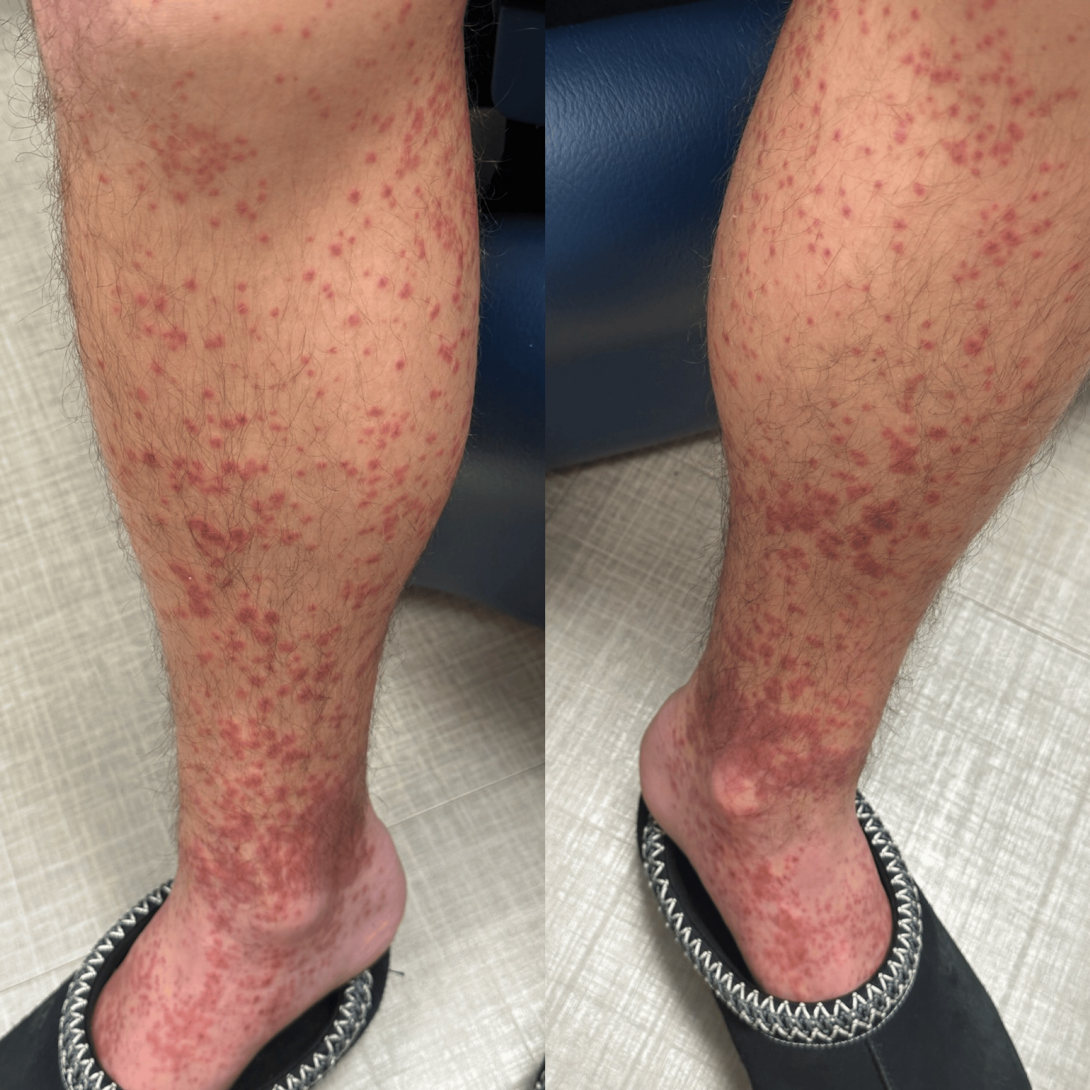
Petechial rash on bilateral legs at the initial clinic visit.

On physical examination, he exhibited posterior oropharyngeal erythema and a non-blanching petechial rash on his arms and legs (Figure 1). The rash was not observed on his palms, soles, back, or face. 

An abdominal ultrasound was ordered to assess for splenomegaly, along with comprehensive blood work. The laboratory tests included a comprehensive metabolic panel, urinalysis, EBV panel, VCA, early antigen-diffuse (EAD) IgG antibody, cytomegalovirus (CMV) IgG and IgM antibodies, CBC with differential, HIV, syphilis antibody, *Chlamydia trachomatis, Neisseria gonorrhoeae,* and a hepatitis panel. All results were negative except for an elevated C-reactive protein (CRP), a positive EBV VCA-IgG, and an elevated WBC count (Table 1). 

**Table 1:** Tests ordered, patient values, and reference ranges.

Parameters	Patient values	Reference range
White blood cell count	10.7 B/L	3.7-10.5 B/L
Platelet count	336 B/L	150-400 B/L
C-reactive protein	2.9 mg/dL	≤0.50 mg/dL
Epstein-Barr virus e​arly antigen Ab, IgG	<0.9 U/mL	0-0.89 U/mL
EBV viral capsid Ag Ab, IgG	162 U/mL	0.0-17.9 U/mL
Cytomegalovirus IgG	<0.60 U/mL	0.0-0.59 U/mL
Cytomegalovirus IgM	<0.30 U/mL	0.0-0.29 U/mL
HIV screen fourth generation	Non-reactive	Non-reactive
Treponema pallidum antibody	Non-reactive	Non-reactive
Chlamydia trachomatis	Negative	Negative
Neisseria gonorrhoeae	Negative	Negative

Later that evening, after leaving the clinic, the patient experienced worsening polyarthralgias, most likely due to IM, which significantly impaired his ability to ambulate. He presented to the emergency department (ED), where he underwent an abdominal ultrasound and was evaluated by an infectious disease (ID) specialist. Ultrasound imaging revealed a spleen with homogeneous echogenicity, measuring 12.9 x 13 x 4.7 cm with an estimated volume of 414.5 cc, consistent with mild splenomegaly. As the ED visit occurred out of network, we were unable to obtain the ultrasound images, only the reported dimensions. The ID specialist concluded that the rash was most likely attributable to acute EBV infection. The patient was advised to rest, avoid contact sports, and return for a follow-up ultrasound in a few weeks.

The patient returned to the clinic 10 days after his initial presentation. His rash had significantly improved without the need for treatment. Unfortunately, a photo could not be uploaded to the electronic medical record (EMR) at that time due to a system outage. Additionally, his RUQ and LUQ abdominal discomfort had resolved. As the patient is an athlete, he was advised to continue refraining from sports activities until a follow-up ultrasound in four weeks. At that subsequent appointment, he was cleared to resume athletic participation based on normal imaging findings.

## Discussion

This patient presented with a petechial rash that developed concurrently with symptoms of IM. The rash was determined to be associated with acute mononucleosis infection. This case is notable, as EBV-associated rashes are more commonly linked to antibiotic use, particularly penicillin. Such rashes are rare, occurring in 2%-3% of patients [11,12]. While the exact mechanism of EBV-related penicillin rashes remains unclear, one hypothesis is that the rash occurs due to a transient loss of immune tolerance. Most affected individuals do not have a known penicillin allergy; however, a retrospective study found that some patients may become sensitized to beta-lactams in the future, which may indicate an allergy. Penicillin-associated rashes are typically described as maculopapular exanthems, which can appear anywhere on the body but frequently involve the palms and soles [13]. Our patient developed a petechial rash without recent exposure to penicillin. 

Petechial rashes have been observed in IM patients; however, they are usually found on the hard or soft palate. Oftentimes, they may coalesce into one. This has been seen in patients who have co-infection with CMV [12]. In this case, the patient tested negative for CMV antibodies and exhibited petechial lesions only on his arms and legs. This effectively rules out CMV-associated petechial rash as a contributing factor.

Given the patient's acute IM, immune thrombocytopenic purpura (ITP) was considered in the differential diagnosis. Several studies have suggested a potential association between IM and ITP. EBV may induce the formation of antiplatelet antibodies through molecular mimicry, which may trigger an autoimmune reaction leading to ITP [14]. However, in this case, the patient exhibited an extensive petechial rash despite having a normal platelet count, effectively ruling out ITP and other thrombocytopenic causes. 

Our patient had also taken a course of azithromycin for *Streptococcus pyogenes* approximately one month prior to presentation. There have been a few reports of petechial or purpuric rashes associated with azithromycin, typically occurring around seven days after administration. Although rare, Stevens-Johnson syndrome and drug reaction with eosinophilia and systemic symptoms (DRESS) have also been reported [15]. However, given the time interval between azithromycin use and rash onset, a drug reaction related to azithromycin was considered unlikely in this case.

EBV has also been associated with various autoimmune diseases, such as systemic lupus erythematosus, Sjögren's syndrome, rheumatoid arthritis, and multiple sclerosis. Although the exact mechanism remains unclear, proposed pathways include immune evasion, lytic reactivation, molecular mimicry, and B cell reprogramming [16]. High titers of anti-EBV antibodies have been observed in patients with these autoimmune diseases. EBV has also been detected in the salivary glands of patients with Sjögren's syndrome and the synovial tissue of individuals with rheumatoid arthritis [17]. In this case, the patient had no history of an autoimmune disorder, making this etiology less likely. However, an autoimmune panel may have given us more insight into the potential early onset of an autoimmune disease. 

Although cutaneous manifestations of EBV in the setting of IM have been reported, they remain relatively uncommon, occurring in approximately 5% of patients. These manifestations include petechial, macular, scarlatiniform, urticarial, and erythema multiforme rashes [18]. In many cases, these dermatologic findings can be attributed to recent penicillin use, CMV co-infection, or ITP. When evaluating rash presentations, it is essential to rule out life-threatening conditions as part of a comprehensive assessment.

## Conclusions

Although petechial rashes following IM have been documented, most cases are attributed to other causes, such as recent antibiotic use or co-infection with CMV or ITP. This case report presents a severe, widespread petechial rash following IM, without the typical contributing factors. These findings highlight the need for further research into the cutaneous manifestations of EBV infection. A limitation of this report is the fragmented clinical documentation, as the patient was seen by multiple providers over a two-week period, including some who were out of network. Additionally, an autoimmune panel might have provided further insight into the underlying etiology of the patient's petechial rash. Physicians to be aware of atypical cutaneous manifestations that can occur with IM to ensure accurate diagnosis and appropriate management.
